# Tool Use and Generalized Motor Programs: We All Are Natural Born *Poly-Dexters*

**DOI:** 10.1038/s41598-018-28759-2

**Published:** 2018-07-11

**Authors:** François Osiurak, Mathieu Lesourd, Ludovic Delporte, Yves Rossetti

**Affiliations:** 1 Laboratoire d’Etude des Mécanismes Cognitifs, Université de Lyon, 5, avenue Pierre Mendès-France, 69676 Bron Cedex, France; 2 Institut Universitaire de France, 103, Boulevard Saint-Michel, 75005 Paris, France; 3 Integrative, Multisensory, Perception, Action, & Cognition Team, Centre de Recherche en Neurosciences de Lyon, INSERM-CNRS-Université de Lyon, 16, avenue Doyen Lépine, 69676 Bron Cedex, France; 4 Mouvement, Handicap et Neuro-Immersion, Hospices Civils de Lyon et Centre de Recherche en Neurosciences de Lyon, Hôpital Henry Gabrielle, 20, route de Vourles, 69230 St Genis Laval, France

## Abstract

For most people, human tool use is inextricably entwined with manual dexterity. This folk belief is widespread among scientists too. In this line, human tool use is based on motor programs about how the hand interacts with tools, implying that the use of end-effectors other than the hand should generate motor control difficulties (e.g., inability to reproduce a specific tool-use action over time), because these so-called programs characterize the spatiotemporal parameters of hand movements, but not of other end-effectors. To test this, we asked participants to perform three tool-use actions (e.g., pounding a nail) with four end-effectors (i.e., right foot, right elbow, left hand, right hand). We show that participants not only spontaneously performed the tool-use actions effectively, but also crucially kept tools’ spatiotemporal parameters constant among the end-effectors. This phenomenon, which we call poly-dexterity, is at odds with the view that the human brain stores hand-centered motor programs for tool use. Poly-dexterity is instead consistent with the idea that, once the tool-use action is formed mentally, general motor programs can be applied to a variety of end-effectors. Reversing the usual evolutionary perspective, our findings support that, in the course of evolution, manual dexterity has come after tool-use skills.

## Introduction

While initially designed for primate arboreal locomotion^1^, the function of the hand progressively evolved in our ancestors, allowing us to develop manual dexterity useful for object-object manipulation and tool use (i.e., exaptation^2^). For some authors, it is the accuracy of manual actions uniquely afforded by the human hand that explains why tool use skills remain limited in the animal kingdom^2,3^. Along the same lines, it has been assumed that this high dexterity results not only from morphological modifications (e.g., opposable thumb^2^), but also from changes within the brain^4^. In humans, the left inferior parietal cortex is assumed to store specific motor programs containing information about how the hand interacts with tools (e.g., for a hammer, a broad oscillation and a power grip^4–11^). The focus is heavily placed on the hand, corroborating the folk belief that tool use is synonymous with manual, but not intellectual work. In broad terms, this manipulation-based approach advocates that human tool use might be unique because of the progressive development of manual dexterity – notably based on hand-centered motor programs (hereafter referred to as the hand-centered motor program hypothesis).

The manipulation-based approach is somewhat inconsistent with the frequent observation that individuals born without arms can nevertheless use many tools with their feet in everyday life (e.g., eating with a fork). While this observation may be interpreted as evidence that manual dexterity is not a prerequisite for tool use, it could also be argued that such atypical behavior results from idiosyncratic neural plasticity and/or extended practice using uncommon end-effectors. This suggests that, when an individual is born without arms, the tool-specific motor programs usually used by ordinary people to specify the hand-centered spatiotemporal parameters are ontogenetically recycled for the use of feet^12^. If this holds true, then in ordinary people this recycling process should not take place spontaneously (e.g., from the first attempt to use feet as end-effectors), but should require a certain amount of practice to enable brain plasticity, perhaps weeks or months^13^. After all, according to the hand-centered motor program hypothesis, tool-use motor programs characterize the spatiotemporal parameters of hand movements, but not those of other potential end-effectors. Therefore the use of end-effectors other than the hand should generate substantial motor control difficulties (e.g., inability to reproduce a specific tool-use action over time). In other words, tools’ spatiotemporal parameters should not be kept constant when ordinary people use tools for the very first time with different uncommon end-effectors.

**Figure 1:**
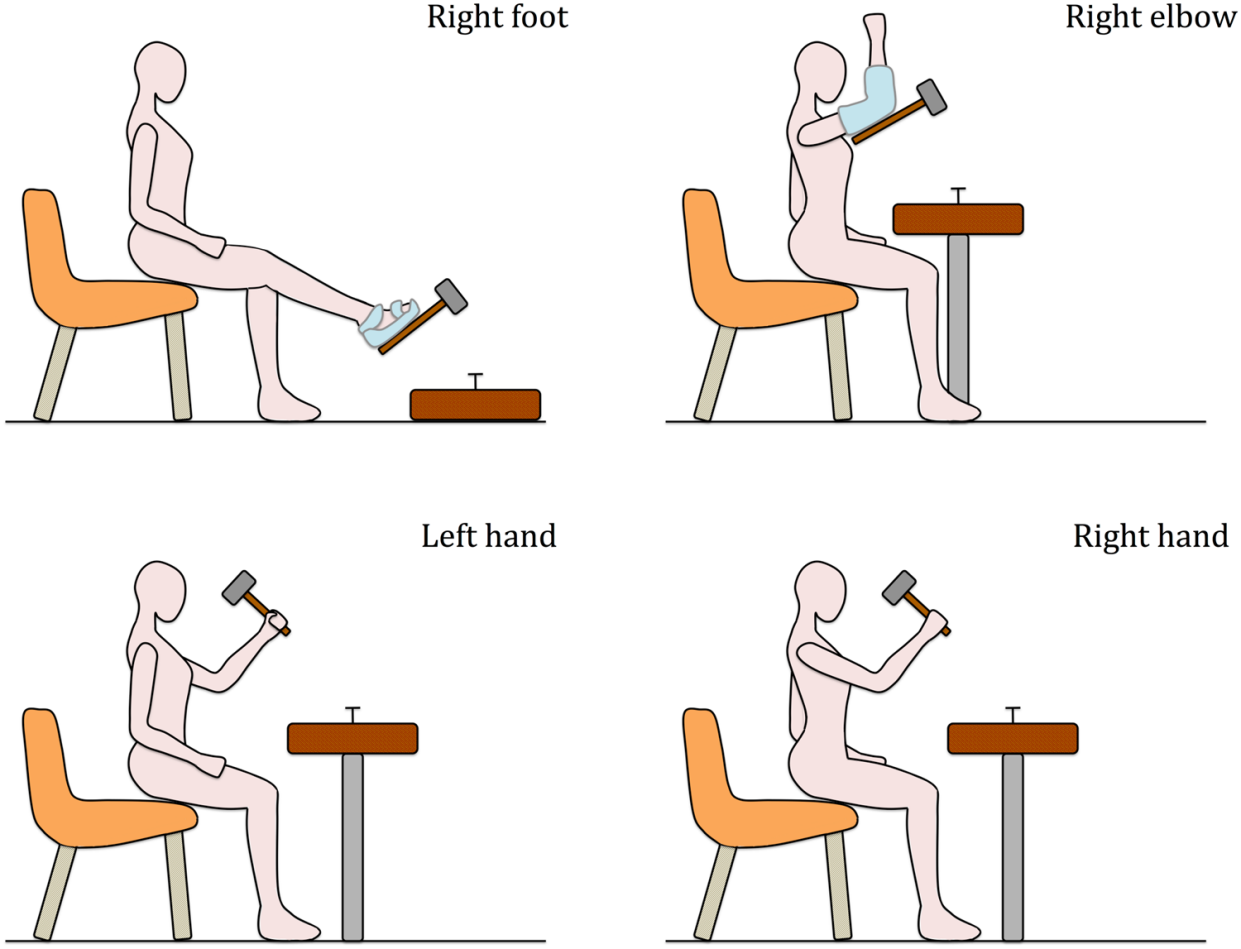
Experimental setup. Ten right-handed participants were asked to perform three real tool-use actions (i.e., pounding a nail with a hammer, sweeping sawdust with a brush, painting with a roller) by using four different end-effectors in the following order: Right foot (customized snowboard fixation), right elbow (customized moldable plastic cast), left hand, and right hand. Here, only the hammer condition is shown.

Here we sought to examine this hypothesis by asking 10 right-handed healthy participants to perform three real tool-use actions (i.e., pounding a nail with a hammer, sweeping sawdust with a brush, painting with a roller) by using four different end-effectors in the following order: Right foot, right elbow, left hand, and right hand, i.e., from the least to the most frequently used. The experimental 3D motion analysis setup used to record the tools’ spatiotemporal parameters is depicted in Fig 1. Our analyses focused on four classical kinematic landmarks^14,15^ (Cycle duration; Maximum velocity of the forth movement; Peak acceleration of the forth movement; Peak deceleration of the forth movement) and one relevant spatial attribute (Vertical movement amplitude).

## Results

**Figure 2:**
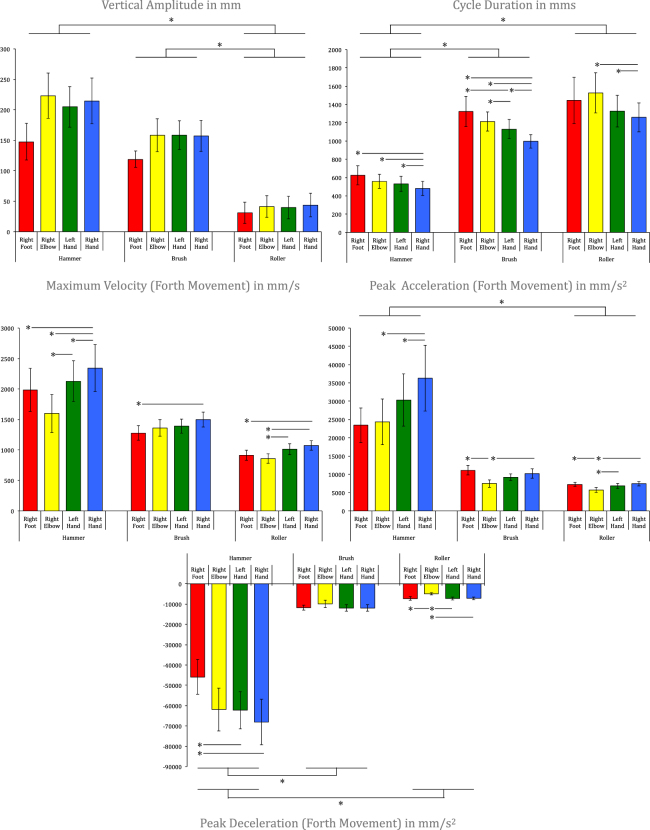
Spatiotemporal parameters of interest in function of tools and end-effectors. For each parameter, a one-way MANOVA with Tool as within-subject factor was conducted on the values obtained for the four end-effectors. Significant differences were found for Vertical Amplitude, Cycle Duration, Peak Acceleration, and Peak Deceleration (all *F*(2,27) > 2.07, all *p* < 0.05), but not for Maximum Velocity (*F*(2,27) = 2.04, *p* = 0.06). When significant, post-hoc MANOVA were conducted for pairwise comparisons. Repeated-measure ANOVA with End-Effector as within-subject factor were also computed to compare the four end-effectors for each tool and each parameter. When significant, post-hoc pairwise *t*-tests corrected with a Holm-Bonferroni procedure were computed. Asterisks denote significant differences.

Although participants felt more at ease with the hands than with the elbow or the foot, they were able to perform the three tool-use actions in an effective way, irrespective of the end-effector used. For instance, on average, the nail was pounded approximately 2.3 cm deep into the table, while this performance varied only moderately among the different end-effectors (*M*_*Right foot*_ = 1.6 cm; *SE*_*Right foot*_ = 0.5 cm; *M*_*Right elbow*_ = 2.2 cm; *SE*_*Right elbow*_ = 0.8 cm; *M*_*Left hand*_ = 3.0 cm; *SE*_*Left hand*_ = 1.5 cm; *M*_*Right hand*_ = 2.5 cm; *SE*_*Right hand*_ = 1.1 cm). A repeated measure ANOVA with End-Effector as within-subject factor revealed that there was no significant difference (*F*(3,27) = 0.47, *p* = 0.71), suggesting that hammering performance was relatively comparable for the different end-effectors. The same was true for the brush and the roller (see Methods Section). Second, as expected, each tool was characterized by specific spatiotemporal parameters, even if these were slightly modified by the biomechanical constraints of each end-effector (e.g., for the roller, peak acceleration was lower for the right elbow than for the other end-effectors; see Fig. 2). The hammer’s motion was ample, with pronounced acceleration and deceleration phases. The roller’s motion was the least ample vertically, was relatively slow, and did not have marked acceleration and deceleration phases. The brush’s motion was relatively similar to roller’s, but with a greater vertical amplitude.

**Figure 3:**
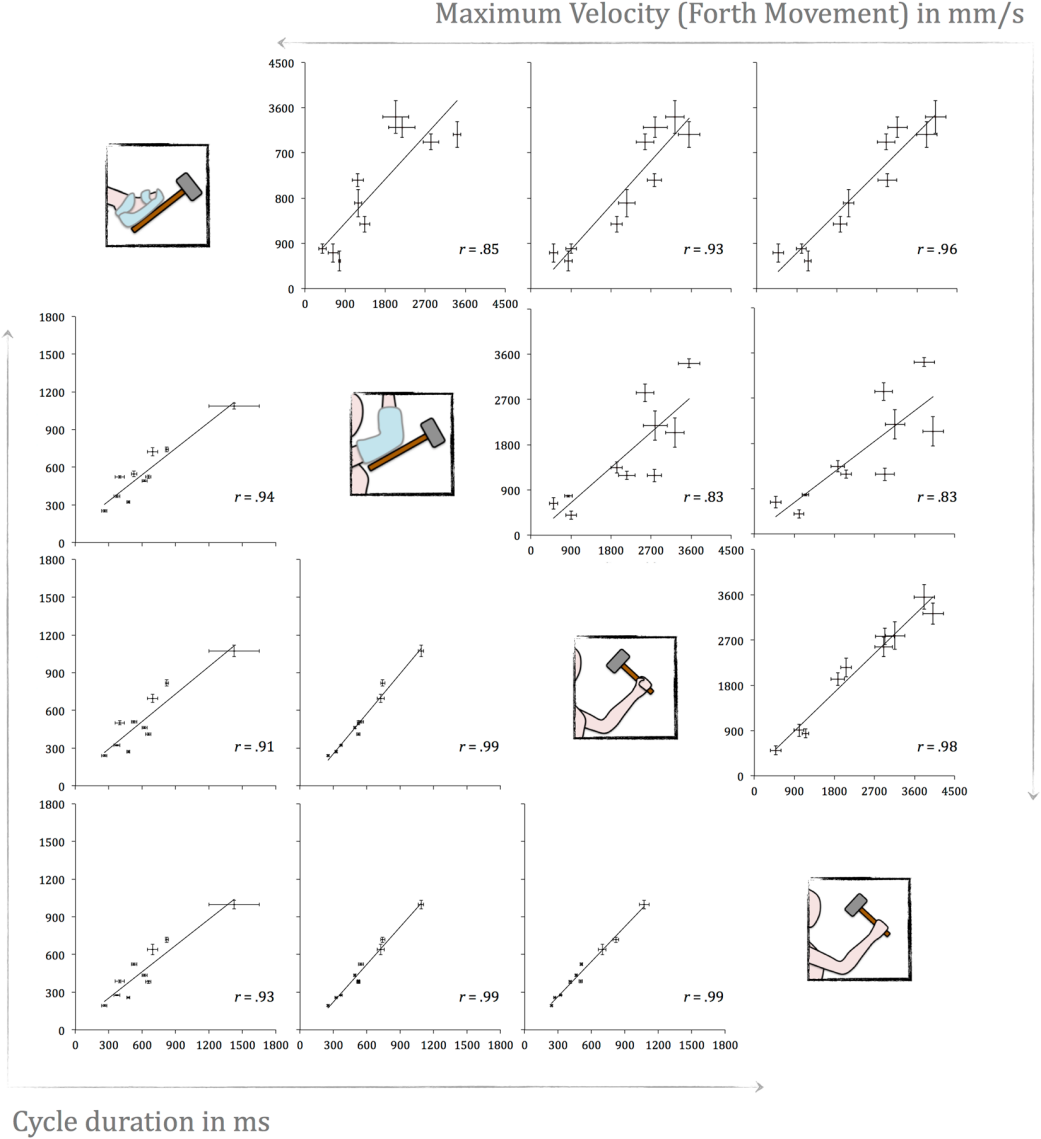
Correlations between the end-effectors for maximum velocity and cycle duration (Hammer condition). Bars represent individual standard errors. The four end-effectors are: Right foot, right elbow, left hand and right hand. Correlations are Pearson’s correlation coefficients. They all are significant (see Table 1).

Let us now turn to the main result of this work. To test the hand-centered motor program hypothesis, we computed correlations between the four end-effectors for each tool and for each parameter of interest. The results are given in Table 1 and an illustration is provided in Fig. 3 for cycle duration and maximum velocity produced by the hammer. Overall, we found highly significant correlations between the end-effectors for each parameter of interest and for each tool (more than 88% of the correlations obtained were statistically significant, with more than 70% above 0.80, and more than 57% above 0.90). The non-significant correlations found (about 12%) were positive and moderate (between 0.43 and 0.68).

**Table 1:** Correlations between end-effectors for each tool and each parameter of interest.

	Hammer				Brush				Roller			
Vertical Amplitude		R-Foot	R-Elbow	L-Hand		R-Foot	R-Elbow	L-Hand		R-Foot	R-Elbow	L-Hand
	R-Elbow	0.68			R-Elbow	0.49			R-Elbow	***0.86***		
	L-Hand	0.63	***0.88***		L-Hand	0.43	***0.93***		L-Hand	***0.76***	***0.98***	
	R-Hand	0.64	***0.85***	***0.98***	R-Hand	0.46	***0.93***	***0.89***	R-Hand	***0.77***	***0.94***	***0.92***
Cycle Duration		R-Foot	R-Elbow	L-Hand		R-Foot	R-Elbow	L-Hand		R-Foot	R-Elbow	L-Hand
	R-Elbow	***0.94***			R-Elbow	***0.89***			R-Elbow	***0.98***		
	L-Hand	***0.91***	***0.99***		L-Hand	***0.94***	***0.98***		L-Hand	***0.99***	***0.98***	
	R-Hand	***0.93***	***0.99***	***0.99***	R-Hand	***0.86***	***0.93***	***0.94***	R-Hand	***0.99***	***0.98***	***0.99***
Maximum Velocity (Forth Movement)		R-Foot	R-Elbow	L-Hand		R-Foot	R-Elbow	L-Hand		R-Foot	R-Elbow	L-Hand
	R-Elbow	***0.85***			R-Elbow	***0.70***			R-Elbow	***0.92***		
	L-Hand	***0.93***	***0.83***		L-Hand	***0.82***	***0.92***		L-Hand	***0.84***	***0.94***	
	R-Hand	***0.96***	***0.83***	***0.98***	R-Hand	***0.74***	***0.81***	***0.83***	R-Hand	***0.87***	***0.95***	***0.93***
Peak Acceleration (Forth Movement)		R-Foot	R-Elbow	L-Hand		R-Foot	R-Elbow	L-Hand		R-Foot	R-Elbow	L-Hand
	R-Elbow	***0.84***			R-Elbow	0.64			R-Elbow	***0.75***		
	L-Hand	***0.82***	***0.96***		L-Hand	0.57	***0.75***		L-Hand	***0.65***	***0.94***	
	R-Hand	***0.87***	***0.96***	***0.98***	R-Hand	0.63	***0.93***	***0.70***	R-Hand	***0.77***	***0.94***	***0.90***
Peak Deceleration (Forth Movement)		R-Foot	R-Elbow	L-Hand		R-Foot	R-Elbow	L-Hand		R-Foot	R-Elbow	L-Hand
	R-Elbow	***0.68***			R-Elbow	***0.62***			R-Elbow	***0.70***		
	L-Hand	***0.86***	***0.78***		L-Hand	***0.96***	***0.75***		L-Hand	***0.84***	***0.82***	
	R-Hand	***0.89***	***0.72***	***0.95***	R-Hand	***0.74***	***0.92***	***0.78***	R-Hand	0.61	***0.86***	***0.80***

R, Right; L, Left; Bold, italicized values are statistically significant. For each matrix, Holm’s correction was applied with an overall *p*-value of 0.05. Correlations are Pearson correlation coefficients (*n* = 10). Additional post-hoc power analyses^38^ were performed: for *r* > 0.80: Power > 0.80; for 0.75 < *r* < 0.80: 0.70 < Power < 0.80; for *r* < 0.75: Power < 0.70.

## Discussion

Our main finding is that participants kept the tools’ spatiotemporal parameters constant among the different end-effectors. For instance, when a participant used the hammer by executing a broader and faster movement compared to other participants, these characteristics were maintained irrespective of the end-effector used (see Fig. S1). This finding is at odds with the hand-centered motor program hypothesis, challenging the popular and widespread idea that the human brain stores specific tool-use motor programs that characterize the spatiotemporal parameters of hand movements^4–11^.

Rather, our results provide support for the reasoning-based approach, which has been recently developed to overcome some limitations of the manipulation-based approach^16–20^. The basic assumption is that tool use is based on the ability to reason about physical object properties, by using mechanical knowledge. This technical reasoning is critical for forming a mental simulation of the tool-use action that constrains the motor actions appropriate to perform the intended tool-use action^16–19^. For instance, if a person aims to powerfully pound a nail, mechanical knowledge is useful to inform her that broad oscillation of the hammer is suitable. However, if the hammering action requires precision (e.g., stone knapping), smaller movements are needed^21^. Neuropsychological and neuroimaging evidence indicates that technical reasoning skills may involve the left inferior parietal cortex^18,22–27^ (particularly the cytoarchitectonic area PF), thus challenging the hypothesis proposed by the manipulation-based approach that this brain area is involved in the storage of hand-centered motor programs.

The important corollary is that we all have the neurocognitive potential to use different end-effectors to perform a given tool-use action, inasmuch as the tool can be grasped and/or manipulated by the end-effector, what we term *poly-dexterity*. Observations of tool use in individuals born without arms already suggest that a high potential for “foot dexterity” can be reached in humans. As mentioned above, according to the manipulation-based approach, this phenomenon could result from extended practice and/or idiosyncratic neural plasticity based on the ontogenetic recycling of hand-centered motor programs. Our findings speak against this hypothesis, by showing that the ability to form a mental simulation of the intended tool-use action can allow people to guide the different end-effectors in a coherent way^28^, even when using uncommon end-effectors for the very first time. As such, the differences between the end-effectors in terms of strength or velocity might be only a matter of biomechanical constraint, but not of motor control per se.

Our findings also support the idea that manual dexterity could have evolved because of the development of specific tool use skills, but not the opposite. Specifically, the fact that people might be natural poly-dexters implies that manual dexterity is not a prerequisite for tool use. Interestingly, the reciprocal is also true, as some left brain-damaged patients encounter severe difficulties when using tools properly even though they have no motor deficit (e.g., motor apraxia^19^). To sum up, the discovery of poly-dexterity described here poses a great challenge to theories that assume that the neurocognitive origins of human tool use lie in the development of specific manual skills.

The issue of motor equivalence is one of the most classical issues in the field of motor control^29,30^, largely explored with the study of handwriting movements. Early reports indicated striking qualitative similarities of writing traces between several end-effectors^29–31^, supporting the generalized motor program hypothesis^32,33^. This hypothesis was tested much later, and challenged, by quantitative kinematic analyses, which failed to provide objective support for these similarities in handwriting movements^34^ or found such evidence only after considerable practice^13^. The present study is the first to address this issue in a context other than such a distinctive skill as handwriting, and our results clearly support the generalized – and not *specific* – motor program hypothesis. As it is obvious that handwriting is one outstandingly practiced tool-use action connected to complex language networks, future research should investigate whether it is motor complexity and/or over practice that reduce the generalizability of motor programs found here with our more representative tool-use actions.

## Methods

### Participants

Ten undergraduate students in cognitive sciences at the University of Lyon (*M*_age_ = 23.4, *SD*_age_ = 2.8; 5 females) took part in the experiment. All participants were right-handed (Edinburgh Score > 70) and had normal or corrected-to-normal visual acuity. Participants had no previous history of neurological or psychiatric illnesses (i.e., motor, perceptual, cognitive or tool-use disorders). In addition, none of them had extensive expertise with the tools chosen for the present experiment (i.e., no professional practice). All participants provided written informed consent. The Ethics Committee of Department of Psychology of Lyon approved the study and the methods were carried out in accordance with the relevant guidelines and regulation.

### Materials and Procedure

Participants were instructed to actually perform three tool-use actions: Pounding a nail with a hammer, sweeping sawdust with a brush, and painting with a roller. We chose these three tool-use actions for the following reasons: (1) To be representative of what is studied in the literature, tool-use actions had to be commonly found in tool-use studies on the topic^22,35,36^; (2) To increase the feasibility of the experiment, tool-use actions had to require large movements; (3) To potentially generalize our results to different tools, we chose to investigate the kinematic parameters of three tool-use actions, and not only of one tool-use action as reported in previous studies on the topic^14,15,37^; (4) To potentially generalize our results to the three dimensional axes, each tool-use action had to be performed preferentially on one of the three axes (i.e. Hammer: Vertical axis; Brush: Lateral axis; Roller: Anteroposterior axis).

The actions had to be carried out with four different end-effectors in the following order: Right foot, right elbow, left hand and right hand. As expected, all participants stated that they never used these types of tools otherwise than with the right hand. No specific instruction was given with regard to how to do the action. For instance, for the hammer, no information was offered about the strength to apply. For each tool, participants had to perform the action with the four end-effectors consecutively, and then another tool was proposed. The order of presentation of tools was counterbalanced between the participants. Participants were told to repeat the action until the experimenter stopped them. They were stopped after 20 to 25 cycles, so that we could obtain 14 cycles corresponding to the cycles 6–19 (i.e., the first five cycles were systematically removed). A cycle corresponded to the total distance travelled by the tool including the forth movement (i.e., for the hammer, the movement from the up position to the nail) and the back movement (i.e., the movement from the nail to the up position).

In all conditions, participants were seated on a chair in front of an adjustable custom table. For the foot condition, the tool was attached under a snowboard binding. For the elbow condition, the tool was attached under a plaster cast requiring to fold the elbow around 90°. For the left hand and the right hand conditions, the tool was grasped by the handle. To make the different end-effector conditions comparable, the handle separating the end-effector (i.e., the base of the thumb, the extremity of the elbow and the extremity of the toes) from the active part of the tool (e.g., the head of the hammer) was made identical for all tools. The table used was one-meter square and 75–90-cm high for the elbow and hands condition. This height was adjusted for each participant in order to make the execution of the action comfortable. For the foot condition, this table was about 10-cm high. For the hammering action, a wooden nail (10-cm length; 2-cm diameter) was placed at the center of the table. The task was to pound it with a wooden hammer. For the brushing action, the tabletop had a 40-cm strip made of glued sawdust. This strip was in a lateral orientation with respect to the participant’s body. For this action, the sawdust should be swept from the right to the left. For the painting action, the tabletop was divided into three strips, two lateral blue 30-cm strips and one central non-colored 40-cm strip. They were in an antero-posterior orientation with respect to the participant’s body. The task was to paint the non-colored strip. Performance of the hammering action was assessed, by measuring the depth to which the nail was driven. We could not use any indicator to specify the performance for the brush and roller conditions, but all participants performed these actions effectively.

### Motion capture

Tools’ motion was recorded using an eight cameras optoelectronic motion capture system (Motion Analysis, 200 Hz). Six passive markers attached to each tool were recorded continuously during each tool-use action. Computed trajectories were filtered using a Butterworth low pas filter at 10 Hz cut-off frequency.

### Data analysis

The *x*−, *y*−, and *z*-axes corresponded to the anteroposterior, lateral and vertical directions with respect to the participant’s body. The spatial attribute of tool motion was quantified by the vertical amplitude. For the brush and the roller, the lateral and the anteroposterior amplitudes could have been chosen, respectively. However, we did not do so, because the bounds of the table constrained the lateral and the anteroposterior amplitude of movements in all participants, considerably reducing inter-individual variability. The vertical amplitude was nevertheless a good indicator to specify the movements for these two tools. The kinematic features were specified by the cycle duration, the maximum velocity, the peak acceleration, and the peak deceleration. For the three latter features, we focused our analyses on the forth movement because it better characterizes the tool-use action performed than the back movement.

## Electronic supplementary material

Supplementary Information
