# Supplementary material for: Tool Use and Generalized Motor Programs: We All Are Natural Born Poly-Dexters
Source: Sci Rep. 2018 Jul 11;8:10429. doi: 10.1038/s41598-018-28759-2 (PMC6041280; doi:10.1038/s41598-018-28759-2)
Supplement: Supplementary file 1 — Supplementary Information [file 41598_2018_28759_MOESM1_ESM.docx]

***Supplementary Information***

Tool Use and Generalized Motor Programs: We All Are Natural Born *Poly-Dexters*

François Osiurak^1,2,*^, Mathieu Lesourd^1^, Ludovic Delporte^3,4^, and Yves Rossetti^3,4^

**Author affiliations**

^1^Laboratoire d’Etude des Mécanismes Cognitifs, Université de Lyon, 5, avenue Pierre Mendès-France, 69676 Bron Cedex, France.

^2^Institut Universitaire de France, 103, Boulevard Saint-Michel, 75005 Paris, France.

^3^Integrative, Multisensory, Perception, Action, & Cognition Team, Centre de Recherche en Neurosciences de Lyon, INSERM-CNRS-Université de Lyon, 16, avenue Doyen Lépine, 69676 Bron Cedex, France.

^4^Mouvement, Handicap et Neuro-Immersion, Hospices Civils de Lyon et Centre de Recherche en Neurosciences de Lyon, Hôpital Henry Gabrielle, 20, route de Vourles, 69230 St Genis Laval, France

***Corresponding author: François Osiurak**

Complete address: Laboratoire d’Etude des Mécanismes Cognitifs, Université de Lyon, 5, avenue Pierre Mendès-France, 69676 Bron Cedex, France

Phone: +33 636 218 891

Email: francois.osiurak@univ-lyon2.fr

**Figure S1.** Amplitude, cycle duration and maximum velocity in function of end-effectors (Participants 2, 4 and 6). Bars represent individual standard errors. As can be seen, Participant 6 performed ampler and faster hammering movements as compared to Participants 2 and 4. So, despite differences resulting from the biomechanical constraints associated with each end-effector, Participant 6 kept these characteristics relatively constant for the four end-effectors. The same is true for the two other participants.
